# Supplementary material for: Prognostic nomogram integrated with inflammatory marker ratios for assessing in-hospital mortality risk in patients with acute type A aortic dissection
Source: PLoS One. 2025 Oct 6;20(10):e0333023. doi: 10.1371/journal.pone.0333023 (PMC12500101; doi:10.1371/journal.pone.0333023)
Supplement: Attachment — Submitted filename: Response to Reviewers.docx [file pone.0333023.s002.docx]

Dear Editors and Reviewers:

Thank you for your letter and for the reviewers “Prognostic nomogram integrated with inflammatory marker ratios for assessing in-hospital mortality risk in patients with acute type A aortic dissection” (PONE-D-25-13134). Those comments are all valuable and very helpful for

revising and improving our paper. We have studied comments carefully and the responds to the reviewer’s comments are as following:

**Journal requirements:**

When submitting your revision, we need you to address these additional requirements.

1. Please ensure that your manuscript meets PLOS ONE's style requirements, including those for file naming. The PLOS ONE style templates can be found at

https://journals.plos.org/plosone/s/file?id=wjVg/PLOSOne_formatting_sample_main_body.pdf and

https://journals.plos.org/plosone/s/file?id=ba62/PLOSOne_formatting_sample_title_authors_affiliations.pdf

**Response:** Thank you for your feedback. We have carefully revised the manuscript to ensure full compliance with PLOS ONE’s style requirements. Please let us know if further adjustments are needed.

2. Thank you for stating the following financial disclosure: “This work was supported by the following grants: L.X.C was funded by Joint Funds for the innovation of science and Technology, Fujian province (No. 2023Y9155). L.X.C was funded by Fujian Provincial Natural Science Foundation of China (NO.2024J01123691). C.L.W received grant from Fujian Provincial Center for Cardiovascular Medicine Construction Project (NO.2128300201).”

Please state what role the funders took in the study. If the funders had no role, please state: "The funders had no role in study design, data collection and analysis, decision to publish, or preparation of the manuscript."

If this statement is not correct you must amend it as needed.

Please include this amended Role of Funder statement in your cover letter; we will change the online submission form on your behalf.

**Response:** Thank you for your comment. We confirm that the funders had no role in the study design, data collection and analysis, decision to publish, or preparation of the manuscript. The funding statements in the cover letter and manuscript have been amended accordingly.

3. We note that your Data Availability Statement is currently as follows: [All relevant data are within the manuscript and its Supporting Information files.]

Please confirm at this time whether or not your submission contains all raw data required to replicate the results of your study. Authors must share the “minimal data set” for their submission. PLOS defines the minimal data set to consist of the data required to replicate all study findings reported in the article, as well as related metadata and methods (https://journals.plos.org/plosone/s/data-availability#loc-minimal-data-set-definition).

For example, authors should submit the following data:

- The values behind the means, standard deviations and other measures reported;

- The values used to build graphs;

- The points extracted from images for analysis.

Authors do not need to submit their entire data set if only a portion of the data was used in the reported study.

If your submission does not contain these data, please either upload them as Supporting Information files or deposit them to a stable, public repository and provide us with the relevant URLs, DOIs, or accession numbers. For a list of recommended repositories, please see https://journals.plos.org/plosone/s/recommended-repositories.

If there are ethical or legal restrictions on sharing a de-identified data set, please explain them in detail (e.g., data contain potentially sensitive information, data are owned by a third-party organization, etc.) and who has imposed them (e.g., an ethics committee). Please also provide contact information for a data access committee, ethics committee, or other institutional body to which data requests may be sent. If data are owned by a third party, please indicate how others may request data access.

**Response:** Thank you for your note. We confirm that all raw data required to replicate the study findings are included in the manuscript. The minimum data set is available in the following file: data.xlsx, and it has been uploaded as a Supporting Information file. We have updated the Data Availability Statement in the manuscript to: "All the data are within the manuscript and its Supporting information files." Please let us know if further clarification is needed.

4. Your ethics statement should only appear in the Methods section of your manuscript. If your ethics statement is written in any section besides the Methods, please delete it from any other section.

**Response:** I would like to thank the reviewers for their valuable advice. We have removed ethics statements from other sections besides the Methods.

**Additional Editor Comments:**

Thank you for submitting your manuscript. After careful evaluation, we have received two sets of reviewer comments, and both reviewers recommend major revision.

Key issues raised include:

1. Concerns about the statistical analysis, specifically the use of discretized variables instead of continuous ones, lack of multicollinearity assessment, unclear significance thresholds and justification for the 7:3 training/validation split.

**Response:** Thank you for the reviewers' comments. The reviewers provided professional opinions on statistical analysis, which were helpful for the improvement of our research. First, regarding the assessment of multicollinearity, according to the conventional analysis process, all variables entering the multifactor analysis can only be qualified if they have undergone multicollinearity testing. At the beginning of writing the article, our team conducted an assessment of multicollinearity, and the collinearity of all variables entering the multifactor analysis was acceptable. We considered this to be part of the regular process, so we did not report on this content. We understand the concerns of the reviewers and editors, and therefore we supplemented this content in the statistical analysis, results, and discussion sections. Secondly, the 7:3 ratio is the empirical segmentation adopted by most clinical research prediction model studies, so we referred to this empirical ratio. Thank you for your comments.

2. Questions regarding biomarker selection criteria (AUC > 0.6), unclear reporting of the postoperative ventilation time cutoff, potential multicollinearity between biomarkers and the need for a more thorough discussion of study limitations (retrospective, single-center design) and external validation.

**Response:** Thank you for the reviewer's comments, which are helpful for the improvement of our research. As can be seen from the article, this is a process of gradually screening variables. In the first round of screening, we used ROC to screen all biomarkers with the aim of selecting variables that are relatively related to the outcome, as well as choosing the best cutoff value, thus selecting the value of 0.6. This is because it is not very strict but still has distinguishing ability. On the other hand, the limitations of the first edition may not be satisfactory, so we have re-discussed the limitations of this study. Thank you for your comments.

3. We ask that you address these points thoroughly in your revised submission. Please provide detailed responses to each comment and make the necessary changes to the manuscript.

**Response:** Thank you very much for your comments. We will address each point thoroughly in our revised submission and provide detailed responses to each comment, along with the necessary changes to the manuscript.

**Reviewers' comments:**

1. Is the manuscript technically sound, and do the data support the conclusions?

The manuscript must describe a technically sound piece of scientific research with data that supports the conclusions. Experiments must have been conducted rigorously, with appropriate controls, replication, and sample sizes. The conclusions must be drawn appropriately based on the data presented.

Reviewer #1: Partly

Response #1:Thank you for the reviewers' comments.

Reviewer #2: Partly

Response #2:Thank you for the reviewers' comments.

2. Has the statistical analysis been performed appropriately and rigorously?

Reviewer #1: Yes

Response #1: Thanks

Reviewer #2: Yes

Response #2: Thanks

3. Have the authors made all data underlying the findings in their manuscript fully available?

The PLOS Data policy requires authors to make all data underlying the findings described in their manuscript fully available without restriction, with rare exception (please refer to the Data Availability Statement in the manuscript PDF file). The data should be provided as part of the manuscript or its supporting information, or deposited to a public repository. For example, in addition to summary statistics, the data points behind means, medians and variance measures should be available. If there are restrictions on publicly sharing data—e.g. participant privacy or use of data from a third party—those must be specified.

Reviewer #1: Yes

Response #1: Thanks

Reviewer #2: Yes

Response #2: Thanks

4. Is the manuscript presented in an intelligible fashion and written in standard English?

PLOS ONE does not copyedit accepted manuscripts, so the language in submitted articles must be clear, correct, and unambiguous. Any typographical or grammatical errors should be corrected at revision, so please note any specific errors here.

Reviewer #1: Yes

Response #1: Thanks

Reviewer #2: Yes

Response #2: Thanks

5. Review Comments to the Author

Please use the space provided to explain your answers to the questions above. You may also include additional comments for the author, including concerns about dual publication, research ethics, or publication ethics. (Please upload your review as an attachment if it exceeds 20,000 characters)

Reviewer #1: This manuscript aims to evaluate the predictive value of preoperative inflammatory biomarker ratios for adverse postoperative outcomes in AAD patients and to construct a prognostic nomogram model to guide anti-inflammatory treatment. My comments on statistical analysis part are given below.

(1) Why not use continuous variable directly in the analysis? Logistic regression handles continuous variable very well. Discretization (e.g. use cut-off point to group continuous values into two groups) will discard information contained in the original continuous variable and thus will lose efficiency in, say, prediction.

**Response #1:** We appreciate the reviewers' comments, which are very professional. After discussion, our team believes that it is necessary to maintain the original results, as the ultimate goal of this study is clinical application. A specific value as the basis for grouping is more convenient in clinical practice. Thank you for your comments.

(2) Correlation between covariates should be examined. In case of co-linearity, one needs to keep one of the co-linear pair. These steps will influence the final results and conclusions.

**Response #1:** Thank you for the reviewers' comments. The reviewers provided professional opinions on statistical analysis, which were helpful for the improvement of our research. First, regarding the assessment of multicollinearity, according to the conventional analysis process, all variables entering the multifactor analysis can only be qualified if they have undergone multicollinearity testing. At the beginning of writing the article, our team conducted an assessment of multicollinearity, and the collinearity of all variables entering the multifactor analysis was acceptable. We considered this to be part of the regular process, so we did not report on this content. We understand the concerns of the reviewers and editors, and therefore we supplemented this content in the statistical analysis, results, and discussion sections. Thank you for your comments.

(3) In multivariate Logistic regression, interactions may be included.

**Response #1:** Thank you for the reviewers' comments.We understand the concerns of the reviewers, and therefore we supplemented the results of the collinearity test.

(4) Please explain why some tests use 0.05 as the significance level while others use 0.1.

**Response #1:** We appreciate the reviewer's insightful question regarding the rationale behind varying significance thresholds (α=0.05 vs. α=0.1). In our study, a significance level of α=0.1 was selected for specific analyses involving small-to-moderate sample sizes to mitigate the risk of Type II errors (i.e., failing to detect true effects due to limited statistical power). This approach aligns with methodological recommendations for exploratory analyses or pilot studies, where overly stringent thresholds (e.g., α=0.05) may overlook clinically meaningful associations in resource-constrained settings. The model we constructed using 0.1 threshold also demonstrated good discrimination. Thank you for your comments.

5. Please explain why $7:3$ is used as the training sample to validation sample ratio. Will such a ratio cause over-fitting problem?

Reviewer #2: The manuscript addresses an important clinical issue with relevant biomarker analyses. However, several aspects require improvement. Firstly, the selection criteria for biomarkers appear arbitrary (AUC > 0.6), lacking robust justification. Clearly define the rationale behind this threshold or consider adjusting based on clinical relevance. Secondly, the postoperative ventilation cutoff (86.5 min) seems unusually brief clinically; clarify if units were misreported (minutes vs hours). Additionally, multicollinearity between biomarkers such as WBC/PLT and Ne/PLT is possible; include correlation analysis or justify their simultaneous use. Lastly, discuss potential biases due to retrospective single-center design and validate externally if feasible. Recommendation: revise.

(1) Please explain why $7:3$ is used as the training sample to validation sample ratio. Will such a ratio cause over-fitting problem?

**Response #2:** We thank the reviewer for raising this critical methodological consideration. The rationale for adopting a 7:3 training-validation split in our study is twofold. Firstly, with a limited sample size (n<1000), a 7:3 ratio ensures sufficient training data for robust parameter estimation while retaining a meaningful subset for preliminary validation. This approach balances the dual objectives of model learning and performance assessment under resource constraints. Secondly, consistent with prior studies[1,2], we employed a 7:3 ratio, adhering to this empirical convention. While split ratios influence validation reliability, overfitting is predominantly governed by: sample size adequacy, model complexity and data quality. Thank you for your comments.

(2) Firstly, the selection criteria for biomarkers appear arbitrary (AUC > 0.6), lacking robust justification. Clearly define the rationale behind this threshold or consider adjusting based on clinical relevance.

**Response #2:** Thank you for the reviewer's comments, which are helpful for the improvement of our research. As can be seen from the article, this is a process of gradually screening variables. In the first round of screening, we used ROC to screen all biomarkers with the aim of selecting variables that are relatively related to the outcome, as well as choosing the best cutoff value, thus selecting the value of 0.6. This is because it is not very strict but still has distinguishing ability. Thank you for your comments.

(3) Secondly, the postoperative ventilation cutoff (86.5 min) seems unusually brief clinically; clarify if units were misreported (minutes vs hours).

**Response #2:** We sincerely appreciate the reviewer's insightful observation regarding the mechanical ventilation duration. The original unit was indeed reported incorrectly. We have made the following corrections: The postoperative ventilation cutoff value has been revised from 86.5 minutes to 86.5 hours throughout the manuscript (including Abstract, Results, Discussion, Conclusion, Table 2 and Table 3). We reconfirmed the raw data from anesthesia records and ICU flow sheets to ensure accuracy. The revised cutoff now appropriately stratifies patients into risk groups for subsequent analyses. These changes have been highlighted in the revised manuscript (Red marked area, Line 38, 156, 167, 168, 243, 265). We thank the reviewer for identifying this critical oversight.

(4) Additionally, multicollinearity between biomarkers such as WBC/PLT and Ne/PLT is possible; include correlation analysis or justify their simultaneous use.

**Response #2:** We sincerely appreciate the reviewers' insightful feedback on the statistical methodology. In response to your comments, we have conducted comprehensive multicollinearity diagnostics (e.g., variance inflation factor [VIF] analysis) to address potential covariate interdependencies. Detailed results are presented in Table 3 (Revised Manuscript). We thank the reviewer for identifying this critical oversight.

(5) Lastly, discuss potential biases due to retrospective single-center design and validate externally if feasible.

**Response #2:** We thank the reviewer for highlighting the limitations associated with our retrospective single-center design. Based on the available data, we have refined the clinical model as comprehensively as possible. In the revised manuscript, we have explicitly acknowledged these limitations in the Discussion section. To address these concerns, future work will include external validation and prospective studies to refine the model.We thank the reviewer for identifying this critical oversight.

**References**

[1] Lin GS, Lu J, Lin J, et al. Value of the Preoperative D-Dimer to Albumin Ratio for Survival and Recurrence Patterns in Gastric Cancer. Ann Surg Oncol. 2023 Feb;30(2):1132-1144. doi: 10.1245/s10434-022-12625-7.
[2] Yu C, Wu X, Zhang S, et al. Prognostic model for survival in patients with neuroendocrine carcinomas of the cervix: SEER database analysis and a single-center retrospective study. PLoS One. 2024 Jan 5;19(1):e0296446. doi: 10.1371/journal.pone.0296446.
