# Supplementary material for: Prognostic nomogram integrated with inflammatory marker ratios for assessing in-hospital mortality risk in patients with acute type A aortic dissection
Source: PLoS One. 2025 Oct 6;20(10):e0333023. doi: 10.1371/journal.pone.0333023 (PMC12500101; doi:10.1371/journal.pone.0333023)
Supplement: Attachment — Submitted filename: Response_to_Reviewers_auresp_2.docx [file pone.0333023.s003.docx]

Dear Editors and Reviewers:

Thank you for your letter and the reviewers' comments on our manuscript "Prognostic nomogram integrated with inflammatory marker ratios for assessing in-hospital mortality risk in patients with acute type A aortic dissection" (PONE-D-25-13134). We sincerely thank the reviewer for their comments in this second round. We provide the following responses to the reviewer's points and respectfully request the editor's careful consideration of our explanations.

**Reviewers' comments:**

1. If the authors have adequately addressed your comments raised in a previous round of review and you feel that this manuscript is now acceptable for publication, you may indicate that here to bypass the “Comments to the Author” section, enter your conflict of interest statement in the “Confidential to Editor” section, and submit your "Accept" recommendation.

**Reviewer #2:** All comments have been addressed.

**Response #2:** Thanks.

2. Is the manuscript technically sound, and do the data support the conclusions?

The manuscript must describe a technically sound piece of scientific research with data that supports the conclusions. Experiments must have been conducted rigorously, with appropriate controls, replication, and sample sizes. The conclusions must be drawn appropriately based on the data presented.

**Reviewer #2:** Partly

**Response #2:** Thank you for the reviewers' comments.

3. Has the statistical analysis been performed appropriately and rigorously?

**Reviewer #2:** Yes

**Response #2:** Thanks.

4. Have the authors made all data underlying the findings in their manuscript fully available?

The PLOS Data policy requires authors to make all data underlying the findings described in their manuscript fully available without restriction, with rare exception (please refer to the Data Availability Statement in the manuscript PDF file). The data should be provided as part of the manuscript or its supporting information, or deposited to a public repository. For example, in addition to summary statistics, the data points behind means, medians and variance measures should be available. If there are restrictions on publicly sharing data—e.g. participant privacy or use of data from a third party—those must be specified.

**Reviewer #2:** Yes

**Response #2:** Thanks.

5. Is the manuscript presented in an intelligible fashion and written in standard English?

PLOS ONE does not copyedit accepted manuscripts, so the language in submitted articles must be clear, correct, and unambiguous. Any typographical or grammatical errors should be corrected at revision, so please note any specific errors here.

**Reviewer #2:** Yes

**Response #2:** Thanks.

6. Review Comments to the Author

**Reviewer #2:** The revised manuscript shows some improvements (e.g., inclusion of multicollinearity diagnostics and correction of ventilator-time units) but key methodological issues remain. This includes the arbitrary choice of AUC > 0.6 for biomarker selection and dichotomization of continuous predictors, which is poorly justified; such cutoffs discard information and can bias results pubmed.ncbi.nlm.nih.gov. Citing "clinical convenience" alone is not convincing, as the clinical validity of the chosen thresholds is unclear. Potential interactions among predictors were not examined. The mixed use of significance thresholds (α = 0.05 vs. 0.1) is unconventional and should be clarified. Similarly, the fixed 7:3 train–test split lacks strong rationale; cross-validation or bootstrapping might better exploit the limited data. External validation is still absent, leaving generalizability untested. For example, performance metrics (e.g., AUC and calibration) are not reported, so predictive accuracy remains unclear. In its current form, I recommend major revision.

**Response #2:** Thank you for the reviewers' comments. The reviewers provided professional opinions on statistical analysis, which were helpful for the improvement of our research.

(1) Regarding the suggestion to use cross-validation for internal validation to utilize existing data more effectively: This is an excellent suggestion. We have supplemented the manuscript accordingly. We employed 10-fold cross-validation to further evaluate the model. The results demonstrate that the model possesses good discriminative performance, with a calibrated C-index of 0.807.
(2) Regarding the concern about converting continuous biomarker values into binary categorical variables: However, this approach is common practice in many studies. For example, in predictive models for hepatocellular carcinoma (HCC) prognosis, cutoff values for AFP (Alpha-fetoprotein) such as 200, 400, or even 800 ng/mL are frequently used. Numerous studies utilize the cutoff method to transform continuous variables into binary categories when establishing thresholds.
1) Concerning AUC threshold selection: Both thresholds of 0.6 and 0.7 are used in existing research. We opted for 0.6 to ensure a broader range of biomarkers entered the univariate analysis, preventing potentially useful clinical indicators from being overlooked.
2) Clinical validity of the threshold: The AUC value demonstrates the clinical validity of the chosen threshold. An AUC > 0.6 indicates the model's predictive ability is superior to standard random guessing (AUC = 0.5), meaning the variable can differentiate between the two classes to a certain extent. Whether it progresses to the final predictive model depends on the results of subsequent analyses.
3) Interaction between predictors: Potential interactions were also assessed through collinearity diagnostics.
(3) Regarding the choice of variable selection method and data splitting: We selected a classic stepwise selection method, which is widely applied in clinical prediction models. The use of mixed significance thresholds and a fixed 7:3 training-test set split are commonly employed in numerous studies. Therefore, we believe these aspects can be retained without modification (Supporting References: DOI: 10.1007/s00431-022-04761-9; DOI: 10.1186/s40001-023-01255-8; DOI: 10.3389/fimmu.2024.1437980). Please give our viewpoints full consideration. Once again, thank you very much for your comments and suggestions.

Yours sincerely,

Liangwan Chen

Department of Cardiovascular Surgery, Fujian Medical University Union Hospital, Xin-quan Road, Fuzhou, Fujian, 350001, PR China.

Email: chenliangwan@fjmu.edu.cn.
