# Supplementary material for: Prognostic nomogram integrated with inflammatory marker ratios for assessing in-hospital mortality risk in patients with acute type A aortic dissection
Source: PLoS One. 2025 Oct 6;20(10):e0333023. doi: 10.1371/journal.pone.0333023 (PMC12500101; doi:10.1371/journal.pone.0333023)
Supplement: Attachment — Submitted filename: Response_to_Reviewers_auresp_3.docx [file pone.0333023.s004.docx]

Dear Editors and Reviewers:

Thank you for your letter and the reviewers' comments on our manuscript "Prognostic nomogram integrated with inflammatory marker ratios for assessing in-hospital mortality risk in patients with acute type A aortic dissection" (PONE-D-25-13134). We sincerely thank the reviewer for their comments in this third round. We have studied comments carefully and the responds to the reviewer’s comments are as following:

**Additional Editor Comments:** Both the original and an additional reviewer agree that the manuscript is undermined by critical methodological flaws - most notably, the use of data-driven dichotomization of continuous variables via ROC analysis. This practice is statistically invalid, discards information, and introduces bias. The defense of this approach as “common practice” reflects a misunderstanding of core biostatistical principles.

Further concerns include misinterpretation of collinearity checks as interaction testing, lack of internal or external validation, non-compliance with TRIPOD standards, and the use of causal language unsupported by the study design.

A full re-analysis is necessary: continuous modeling of predictors, formal interaction testing, internal validation (e.g., bootstrapping), and adherence to TRIPOD guidelines - as mentioned by the Reviewer 2. Without these changes, the paper cannot be accepted.

**Response:** We sincerely thank the reviewers and editors for their rigorous critique. We fully acknowledge the critical methodological flaws identified, particularly the inappropriate dichotomization of continuous variables and insufficient validation. We unequivocally accept all criticisms and have implemented comprehensive revisions, such as retaining continuous variables in their original form without dichotomization, with detailed measures specified in our point-by-point response to Reviewer 2.

**Reviewers' comments:**

**Comments to the Author**

1. Is the manuscript technically sound, and do the data support the conclusions?

The manuscript must describe a technically sound piece of scientific research with data that supports the conclusions. Experiments must have been conducted rigorously, with appropriate controls, replication, and sample sizes. The conclusions must be drawn appropriately based on the data presented.

**Reviewer #2:** Partly

**Response:**Thank you for the reviewers' comments.

2. Has the statistical analysis been performed appropriately and rigorously?

**Reviewer #2:** No

**Response:**Thank you for your valuable comments on the rigor of statistical analysis. We have carefully reviewed the Methods section, and made point-by-point revisions in response to the reviewers' comments.

3. Have the authors made all data underlying the findings in their manuscript fully available?

The PLOS Data policy requires authors to make all data underlying the findings described in their manuscript fully available without restriction, with rare exception (please refer to the Data Availability Statement in the manuscript PDF file). The data should be provided as part of the manuscript or its supporting information, or deposited to a public repository. For example, in addition to summary statistics, the data points behind means, medians and variance measures should be available. If there are restrictions on publicly sharing data—e.g. participant privacy or use of data from a third party—those must be specified.

**Reviewer #2:** Yes

**Response:**Thanks.

4. Is the manuscript presented in an intelligible fashion and written in standard English?

PLOS ONE does not copyedit accepted manuscripts, so the language in submitted articles must be clear, correct, and unambiguous. Any typographical or grammatical errors should be corrected at revision, so please note any specific errors here.

**Reviewer #2:** No

**Response:** We thank the reviewer for highlighting the language concerns. We have revised the language throughout the manuscript, including correcting grammatical and syntactical errors, to meet PLOS ONE's publication standards.

5. Review Comments to the Author

Please use the space provided to explain your answers to the questions above. You may also include additional comments for the author, including concerns about dual publication, research ethics, or publication ethics. (Please upload your review as an attachment if it exceeds 20,000 characters)

**Reviewer #2:** This manuscript, developing a prognostic nomogram for aortic dissection, is invalidated by fundamental methodological errors. Its primary flaw is dichotomizing continuous predictors using data-dredged "optimal" cut-points from ROC analysis—a statistically invalid practice that discards information and creates bias. The author's reply, defending this as "common practice," reveals a critical misunderstanding of biostatistical principles. The study also incorrectly equates collinearity diagnostics with testing for interactions, lacks external validation, and fails to meet TRIPOD reporting standards. The English is imprecise, using causal language unsupported by the design. A complete re-analysis is mandatory: predictors must be modeled continuously, interactions formally tested, and the model validated using bootstrapping, with full adherence to TRIPOD guidelines.

**Response #2:** We sincerely thank Reviewer #2 for their rigorous and constructive critique, which has highlighted critical methodological shortcomings in our study. We fully acknowledge the validity of the concerns raised. Based on the reviewers' comments and our team's discussion, we have comprehensively revised our manuscript by selecting clinically relevant inflammatory markers including white blood cell count, neutrophil count, hemoglobin, D-dimer, blood urea nitrogen, albumin, and neutrophil-to-lymphocyte ratio; to prevent loss of information, we preserved continuous variables in their original form rather than dichotomizing them (e.g., treating values >50 as equivalent when 51 and 99 represent distinct clinical states); additionally, to avoid multicollinearity, we excluded blood cell parameters used to derive inflammatory indices and calculated variance inflation factors (VIF < 5) for included variables; finally, due to unavailable external validation cohorts, we implemented internal validation through bootstrapping while explicitly acknowledging this limitation in the Discussion. We extend sincere appreciation to the handling editor and reviewers for their guidance.

Once again, thank you very much for your comments and suggestions.

Yours sincerely,

Liangwan Chen

Department of Cardiovascular Surgery, Fujian Medical University Union Hospital, Xin-quan Road, Fuzhou, Fujian, 350001, PR China.

Email: chenliangwan@fjmu.edu.cn.
