# Supplementary material for: Prognostic nomogram integrated with inflammatory marker ratios for assessing in-hospital mortality risk in patients with acute type A aortic dissection
Source: PLoS One. 2025 Oct 6;20(10):e0333023. doi: 10.1371/journal.pone.0333023 (PMC12500101; doi:10.1371/journal.pone.0333023)
Supplement: Attachment — Submitted filename: Response_to_Reviewers_auresp_4.docx [file pone.0333023.s005.docx]

Dear Editors and Reviewers:

Thank you for giving us the opportunity to revise our manuscript [PONE-D-25-13134] entitled “Prognostic nomogram integrated with inflammatory marker ratios for assessing in-hospital mortality risk in patients with acute type A aortic dissection”. We are grateful to the editor and the reviewers for their time and insightful comments, which have helped us to improve the quality of our paper. We have carefully addressed all the points raised in the review.

**Journal Requirements:**

1. If the reviewer comments include a recommendation to cite specific previously published works, please review and evaluate these publications to determine whether they are relevant and should be cited. There is no requirement to cite these works unless the editor has indicated otherwise.

Please review your reference list to ensure that it is complete and correct. If you have cited papers that have been retracted, please include the rationale for doing so in the manuscript text, or remove these references and replace them with relevant current references. Any changes to the reference list should be mentioned in the rebuttal letter that accompanies your revised manuscript. If you need to cite a retracted article, indicate the article’s retracted status in the References list and also include a citation and full reference for the retraction notice.

**Response:** Thank you for the suggestion. We have conducted a thorough review of the reference list to further enhance the academic rigor of our manuscript. During this process, we noted that the reference "[24] Zhang Y, Chen T, Chen Q, Min H, Nan J, Guo Z. Development and evaluation of an early death risk 364 prediction model after acute type A aortic dissection. Ann Transl Med. 2021 Sep;9(18):1442. doi: 365 10.21037/atm-21-4063." (originally cited at [Line 364-366]), while relevant, was not indexed in SCI and its supporting evidence was somewhat limited. To more robustly substantiate our argument and align with the highest scholarly standards, we have replaced it with a more authoritative publication from a SCI-indexed journal: "[24]Moisi MI, Bungau SG, Vesa CM, Diaconu CC, Behl T, Stoicescu M, et al. Framing Cause-Effect Relationship of Acute Coronary Syndrome in Patients with Chronic Kidney Disease. Diagnostics (Basel). 2021 Aug 23;11(8):1518. doi: 10.3390/diagnostics11081518.". This newer reference provides stronger and more compelling evidence for the point discussed, and the change has been incorporated at [Line 373-375] in the revised manuscript.

Furthermore, we have performed a comprehensive check of the entire reference list to ensure its full compliance with the journal's guidelines. This includes verifying the accuracy and completeness of all citations, confirming that no retracted papers are cited, and ensuring that the references are up-to-date and encompass the most relevant literature in the field.

**Review Comments to the Author**

**Reviewer #2:** The authors have implemented the key corrections requested in the earlier rounds. The major concern-ROC-based dichotomization-has now been addressed, and variables are retained in their continuous form. Multicollinearity was formally checked with VIF, and internal validation by bootstrapping was performed, with limitations clearly acknowledged. The revised modeling strategy (Hb, NLR, and MODS) is methodologically sound and presented transparently.

Remaining issues are minor. The paper still uses slightly casual causal phrasing in places (“guiding therapy,” “improving prognosis”), which should be softened to “may assist” or “is associated with.” Also, while external validation is understandably not possible, this limitation should be emphasized again in the discussion or acknowledgments. Finally, English clarity has improved, but residual phrasing errors persist (e.g., “conduct more accurate multi-indicator joint individualized predictions”) that could benefit from professional editing.

Overall, the fatal flaws have been corrected. With minor language polishing and acknowledgment of external validation limits, the manuscript is acceptable.

**Response:** We sincerely thank Reviewer #2 for their positive feedback and acknowledgment that our revisions have addressed the major methodological concerns. We are also grateful for the constructive suggestions regarding language and clarity. We have carefully implemented all remaining recommendations as follows:

(1) Causal phrasing: All instances of overly strong language (e.g., "guiding therapy," "improving prognosis") have been softened throughout the manuscript to more cautious and accurate terms.
(2) Emphasis on external validation limitation: As suggested, we have further emphasized the absence of external validation as a key limitation in the Limitation section.
(3) Professional English editing: The manuscript has been professionally edited for English language by American Journal Experts (Certificate Number: 98BA-E44B-9F71-2820-0439). All suggested changes have been reviewed and incorporated by the authors to ensure clarity and readability.

We thank the reviewer again for their thorough and helpful assessment of our work.

Yours sincerely,

Liangwan Chen

Department of Cardiovascular Surgery, Fujian Medical University Union Hospital, Xin-quan Road, Fuzhou, Fujian, 350001, PR China.

Email: chenliangwan@fjmu.edu.cn.
